# What determines the effects and costs of breast cancer screening? A protocol of a systematic review of reviews

**DOI:** 10.1186/s13643-017-0510-y

**Published:** 2017-06-28

**Authors:** O. Mandrik, O. I. Ekwunife, N. Zielonke, F. Meheus, J. L. Severens, S. K. Lhachimi, R. Murillo

**Affiliations:** 1 Early Detection and Prevention Section, International Agency for Research on Cancer, 150 Cours Albert Thomas, 69372 Lyon, France; 2 Institute of Health Policy & Management, Erasmus University Rotterdam, Burgemeester Oudlaan 50, 3062 PA Rotterdam, The Netherlands; 3 Collaborative Research Group for Evidence-Based Public Health, Department of Prevention and Evaluation, Leibniz Institute for Prevention Research and Epidemiology, BIPS/University of Bremen, Achterstraße 30, 28359 Bremen, Germany; 4 Department of Clinical Pharmacy and Pharmacy Management, Nnamdi Azikiwe University, PMB 5025, Awka, Nigeria; 5 Department of Public Health, Erasmus MC, University Medical Center Rotterdam, ’s-Gravendijkwal 230, 3015 CE Rotterdam, The Netherlands; 6 iMTA, Institute for Medical Technology Assessment, Erasmus University Rotterdam, Burgemeester Oudlaan 50, 3062 PA Rotterdam, The Netherlands; 7 Institute for Public Health and Nursing Research—IPP, Health Sciences Bremen, University of Bremen, Grazer Straße 2, 28359 Bremen, Germany; 8 Centro Javeriano de Oncología, Hospital Universitario San Ignacio, Carrera 7 No 40-62, Bogota, Colombia; 9 Faculty of Medicine, Pontificia Universidad Javeriana, Carrera 7 No. 40 - 62, Bogotá, Colombia

**Keywords:** Breast cancer screening, Systematic review, Costs, Efficacy, Effectiveness, Cost-effectiveness

## Abstract

**Background:**

Multiple reviews demonstrated high variability in effectiveness and cost-effectiveness outcomes among studies on breast cancer screening (BCS) programmes. No study to our knowledge has summarized the current evidence on determinants of effectiveness and cost-effectiveness of the most used BCS approaches or tried to explain differences in conclusions of systematic reviews on this topic. Based on published reviews, this systematic review aims to assess the degree of variability of determinants for (a) effectiveness and (b) cost-effectiveness of BCS programmes using mammography, clinical breast examination, breast self-examination, ultrasonography, or their combinations among the general population.

**Methods:**

We will perform a comprehensive systematic literature search in Cochrane, Scopus, Embase, and Medline (via Pubmed). The search will be supplemented with hand searching of references of the included reviews, with hand searching in the specialized journals, and by contacting prominent experts in the field. Additional search for grey literature will be conducted on the websites of international cancer associations and networks. Two trained research assistants will screen titles and abstracts of publications independently, with at least random 10% of all abstracts being also screened by the principal researcher. The full texts of the systematic reviews will then be screened independently by two authors, and disagreements will be solved by consensus. The included reviews will be grouped by publication year, outcomes, designs of original studies, and quality. Additionally, for reviews published since 2011, transparency in reporting will be assessed using the Preferred Reporting Items for Systematic Reviews and Meta-Analyses (PRISMA) checklist for the review on determinants of effectiveness and a modified PRISMA checklist for the review on determinants for cost-effectiveness. The study will apply the Assessing the Methodological Quality of Systematic Reviews checklist to assess the methodological quality of systematic reviews. We will report the data extracted from the systematic reviews in a systematic format. Meta-meta-analysis of extracted data will be conducted when feasible.

**Discussion:**

This systematic review of reviews will examine the degree of variability in the effectiveness and cost-effectiveness of BCS programmes.

**Systematic review registration:**

PROSPERO CRD42016050764 and CRD42016050765

**Electronic supplementary material:**

The online version of this article (doi:10.1186/s13643-017-0510-y) contains supplementary material, which is available to authorized users.

## Background

Breast cancer screening (BCS) is a complex multidisciplinary approach that aims to reduce mortality from one of the most common causes of cancer-related death among women [1]. Different screening tests have been used for implementing BCS programmes among the general female population including breast self-examination (BSE), clinical breast examination (CBE), ultrasonography (US), and mammography.

Multiple country-level and international recommendations have been developed to guide the implementation of BCS policies [2-6]. However, despite being based on extensive evidence reviews, these documents recommend differently. For example, the International Agency for Research on Cancer and the US Preventive Service Task Force conclude on sufficient evidence regarding the benefits of mammography for 50–74-year-old women [3, 4], the European Society for Medical Oncology (ESMO) [2] for 50–69 years old, the American Cancer Society [5] from the age of 45 and continuing after 69 if life expectancy is more than 10 years, and the UK National Health Service for women aged 50 to 70 years old every 3 years [6]. The other screening modalities are either seen as lacking adequate evidence on mortality reduction by the latest guidelines (for example, BSE and CBE) or mainly recommended for high-risk population groups or as diagnostic tools (for example, the US and magneto-resonance imaging) [2–6].

BCS can achieve a decrease in mortality only if appropriately implemented. But, even though effectiveness (i.e. a mortality decrease) is the main consideration for BCS adoption, cost-effectiveness of screening modalities has become an integral part of the decision-making process. Both clinical and economic evaluations of BCS can be done as a trial or a modelling study. While the former is a time- and budget-consuming method, the latter frequently requires sophisticated data and comprehensive statistics. Since high-quality data related to effectiveness and cost-effectiveness of screening programmes may not be available in a country of interest, the targeted outcomes may be approximated from other jurisdictions. There are multiple reasons why effectiveness and cost-effectiveness of screening interventions might differ between the countries, including variability in implementation characteristics, healthcare capacity and clinical practices, treatment adherences and population preferences, demographic and epidemiologic characteristics, or unit prices. The International Society for Pharmacoeconomics and Outcomes Research Good Research Practices Task Force Report on transferability defined that the parameters are called generalizable if they are applied without adjustment and transferable if they could be adapted to other countries’ settings [7].

A considerable number of systematic reviews have explored the effectiveness of BCS in terms of a reduction in mortality as well as the costs-effectiveness of such programmes. These reviews demonstrated high variability in types and values of outcomes which lead to different conclusions and recommendations on the most appropriate BCS strategy. For example, both reviews from Hamashima et al. [8] and Lee et al. [9] recommended mammography as a BCS modality, while Nelson et al. [10] and Gøtzsche and Jørgensen [11] concluded on small magnitude of benefits from screening mammography comparing to the possible harms.

Despite many reviews evaluating the quality of included studies, to our knowledge, no study has summarized the current evidence on the determinants of effectiveness and cost-effectiveness of routinely used BCS approaches or explored the possible differences in the conclusions of systematic reviews on this topic. It is also under question when the results of these reviews can be applied to other countries of interests, given the variability of data between the countries and the studies. We propose a study synthesizing all systematic reviews published until February 2017 reporting effect and efficiency characteristics of BCS programmes.

## Methods/design

### The aim and objectives

This systematic review aims to assess the degree of variability and transferability of determinants of (a) effectiveness (review 1) and (b) cost-effectiveness (review 2) of BCS programmes using mammography, BSE, CBE, ultrasonography, or their combinations among the general female population.

The specific research objectives are as follows:

1. To summarize the evidence on the determinants of effectiveness and cost-effectiveness of routine BCS programmes
2. To explore the variability in values of the determinants of effectiveness and cost-effectiveness between reviews and countries
3. To explore the coherence of systematic reviews and meta-analyses on the determinants of effectiveness and cost-effectiveness
4. To identify factors explaining the differences in conclusions of the systematic reviews included in this systematic review of reviews, if any observed
5. To explore how the effect of the programme’s characteristics as well as the country’s characteristics on determinants for effectiveness and cost-effectiveness of BCS was investigated in the systematic reviews

### Study design

We designed the protocol in accordance with the Cochrane handbook, personal rationale, and the Preferred Reporting Items for Systematic reviews and Meta-Analyses Protocols (PRISMA-P) checklist (see Additional file 1 for completed checklist) [12, 13]. The protocol of two reviews is registered with the International prospective register of systematic reviews (PROSPERO), the registration numbers are CRD42016050764 (review 1) and CRD42016050765 (review 2) [14].

### Eligibility criteria

The study will include systematic reviews that summarize information on determinants of effectiveness (review 1) and cost-effectiveness (review 2) of BCS. Systematic reviews with any type of comparison group will be included if they:

- Assess the following screening approaches: film or digital mammography, ultrasonography, CBE, or BSE
- Focus on a population of women older than 18 years without symptoms of breast cancer and with an average risk in this disease
- Use a systematic approach for literature search using the preliminary specified inclusion/exclusion criteria
- Conduct a literature search on more than one country
- Provide an individual assessment and systematic presentation of the characteristics and outcomes for the included studies (forest-plot, table, or any other structured data presentation)
- Present the targeted primary or secondary outcomes
- Have a full-text publication

Since resource use and cost of breast cancer defines cost-effectiveness of screening, reviews will be also included if they (1) focus on a female population older than 18 years old, diagnosed with breast cancer at any stage and with average risk of disease progression; (2) include common treatment approaches (containing surgeries, radiation, and pharmacological interventions) used in actual clinical practice to treat treatment-naïve, refractory, and relapsed patients with breast cancer, and (3) assess the relevant primary or secondary outcomes.

We will exclude reviews that focus exclusively or in combination on the following parameters: costs and outcomes of co-morbidities of breast cancer, non-screening technologies such as personalized medicine or any original innovative treatment, non-traditional medicines, hypnosis or psychological support, alternative treatments, herbal treatments, treatment of sleep, sexual or emotional disorders, yoga, or other practices typically not covered by the healthcare system.

The primary outcomes for our review 1 are (1) absolute and relative reduction in breast cancer, cancer, and general mortality and (2) participation rate. Since participation rates are frequently assessed in studies examining interventions aimed at increasing adherence to screening (interventions such as a standard invitation letter, a call, or a combination of invitation techniques), these reviews are also included. The secondary extracted outcomes for review 1 are incremental breast cancer detection rate, size of detected cancers, sensitivity of the screening programmes and the test, recall rate, positive predictive value, number needed to screen, and proportional internal cancer rate.

The primary outcomes for our review 2 are (1) mean direct BCS costs; (2) incremental cost-effectiveness ratio, expressed as the cost per quality adjusted life years in the baseline analysis; (3) incremental cost-effectiveness ratio expressed in cost per life-years gained in the baseline analysis; (4) total costs of breast cancer, and (5) direct costs of breast cancer. The secondary extracted outcomes for review 2 are median and/or mean direct, indirect and total breast cancer and breast cancer screening costs, costs for productivity loss related to breast cancer, costs for informal care related to breast cancer, medical resource use, non-medical resource use, average duration of hospitalization, average number of hospitalizations per patient, average number of working days lost, percentage of women receiving targeted therapy and hormone stimulants, resources used related to breast cancer screening, life-years gained/saved, quality adjusted life years, incremental cost-effectiveness ratio in costs per cost per disability adjusted life years, and cost per death averted.

### Search strategy

A comprehensive systematic search of “reviews” will be conducted in Scopus, Embase, and Medline (via PubMed) databases from inception to February 2017 with no language restrictions. The search strategy for Medline via PubMed is described in an additional document file (see Additional file 2). The keywords “breast cancer AND screening” are searched in the titles and abstracts of reviews of the Cochrane database.

In addition, the search will be augmented by identifying further studies from reference lists of identified relevant studies or reviews, by hand searching in the specialized journals, and by contacting the prominent experts in the field. A search for grey literature will be conducted on the websites of the American Society of Clinical Oncology (ASCO) [15], the American Cancer Society [16], the International Agency for Research on Cancer [17], and the ESMO [18]. The additional search will include the keywords “authors’ names AND breast cancer” in order not to miss the updates of the conducted reviews and search in PROSPERO database to identify if any reviews are currently under development.

### Data management

Systematic reviews obtained from the search are exported to EndNote X8^TM^ (Clarivate Analytics, Philadelphia, USA) where duplicates will be identified and removed. Two trained research assistants will screen all titles and abstracts of publications independently, with at least 10% of all abstracts also screened by the principal researcher (OM). The full texts of publications will be then screened for eligibility independently by the two investigators. All disagreements will be solved by consensus between the reviewers. Native speakers assist with data extraction if an article is written in a language other than English.

A data extraction sheet developed in Microsoft Excel® 2010 will be tested on two randomly selected studies. All data will be extracted by one researcher and confirmed by the second one. From each of the systematic reviews, we will extract the general information (author(s), title, years of publication and systematic search, targeted geographic search, and sponsorship), methods of systematic review applied and methodology of studies included into the reviews, the main reported information (the main outcomes used, finding, conclusions, and limitations), means, standard deviations, and ranges for primary and secondary outcomes.

The references from the included publications will be analysed if information from the systematic reviews is incomplete to fill in the extraction sheet. Each review will be assigned non-exclusively to the groups by (1) outcomes presented, (2) year of publication (all the studies and those published from 2011 and upward), (3) design of the studies included into the reviews, and (4) quality.

The authorship, methods, and results sections of all the included reviews will be explored in details to identify the following:

Duplicates: reviews published in different journals and with possible differences in methods description, additional/sub-group analyses, and/or data, but presenting the same results for the main outcomes. We will include the latest review if a duplicate is identified.

An update of another study: review-publications updating previous reviews or fully including the results of previously published reviews. All updates of the studies are included into data extraction, while only the latest updated review will be combined into the summary of the latest evidence.

Related publications: studies related to each other by time and authorship, but presenting fully or partially different information. Each of the related publications will be included into the analysis while being grouped in clusters to address possible bias.

### Quality appraisal

All included publications will be appraised by two independent reviewers applying the Assessing the Methodological Quality of Systematic Reviews (AMSTAR) checklist and considering selection, reporting, and publication biases [19]. For those studies that have been judged to be of medium or high quality and which were published after 2011, we will additionally assess the transparency of reporting by using PRISMA checklist for review on determinants of effectiveness and an adapted PRISMA checklist to assess transparency of presentation of the results in systematic reviews on costs and cost-effectiveness of breast cancer screening (see Additional file 3) [20]. Studies of medium or high quality published before 2011 will remain for data synthesis only if no other high-quality evidence for this outcome is available. The disagreement between the reviewers on studies’ evaluations will be resolved by consensus and involve a third party if consensus cannot be reached.

### Data analysis

We will use Cohen’s kappa coefficient to define the strength of agreement between the two evaluators. The analyses reported in the included systematic reviews will be extracted and reported in a systematic (table) format together with reviews’ descriptive characteristics and evaluations on their quality. A separate summary on outcomes for each of the analysed subgroups will be provided. We will discuss transferability of data from the systematic reviews considering both quality of the reviews and the included studies, transparency in reporting, geographic presentation in the reviews, variability in outcome values reported by the reviews and systematic differences in outcomes between countries.

Depending on the results of our systematic review of reviews, primarily in relation to data heterogeneity, we will also conduct meta-meta-analyses. For this, we will select the reviews included with the relevant outcomes and code their data for three age categories of women (<49; 50–69; >70). We also aim to assess (1) an impact of the quality of the study on the results of meta-analyses and (2) a difference in outcomes between the designs of studies included into a systematic review. We will apply a fixed effects model for those interventions and population groups where heterogeneity is small and a random effects model when heterogeneity leads to higher *I* ^2^ index considering possible variation in values related to geographic differences in programme implementation, demographic, economic parameters, and healthcare coverage. To address an overlapping of meta-analyses, we will quantify the uniqueness of each of the studies prior to meta-analyse the effect. The homogeneity of effect sizes will be assessed using a *Q* test.

## Discussion

The decrease in mortality from breast cancer is the main purpose of screening programmes. However, an evaluation of the impact of screening must consider the organizational structure of BCS delivery since the screening parameters and populations are very heterogeneous given the differences in contextual factors between countries and significant variations in the determinants of effectiveness and cost-effectiveness in high- and low-income countries. Even with similar performance characteristics (i.e. sensitivity and specificity) of BCS programmes, differences in programme attributes such as demographic and structural parameters will affect both outcomes and cost in different countries. Because of these variations, extrapolation of some of the determinants of effectiveness and cost-effectiveness of BCS (e.g. participation rates, treatment pathways, or unit costs of screening implementation and cancer treatment) is difficult.

It is therefore important to carry out a systematic review of reviews and critically summarize available evidence on factors related to the impact of breast cancer screening on population costs and health outcomes. This comprehensive search is targeted to identify all published reviews and meta-analyses on the effectiveness and cost-effectiveness of BCS in high-, middle-, and low-income countries. It aims at examining the reasons for differences between the reviews’ conclusions and recommendations, to explore how the conclusions of systematic reviews evolved over time and with new evidence and to summarize the latest evidence based on systematic reviews with low risk of bias.

We use Cochrane’s definition of a systematic review [12] and assess the methodological quality of systematic reviews to limit the risk of bias using AMSTAR guidelines [19]. While these choices may limit the number of systematic reviews that will be included in this systematic review of reviews, it guarantees the best available evidence on which we base the conclusions. The result of our systematic review will summarize the available evidence on determinants of effectiveness and costs of breast cancer screening programmes and define the needs for future research.

## Additional files

Additional file 1: PRISMA-P checklist. (DOC 95 kb)

Additional file 2: The search strategy for Medline via PubMed. (DOCX 15 kb)

Additional file 3: An adapted PRISMA checklist to assess transparency of presentation of the results in systematic reviews on costs and cost-effectiveness of breast cancer screening. (DOCX 15 kb)

## Abbreviations

AMSTAR: Assessing the Methodological Quality of Systematic Reviews
BCS: Breast cancer screening
BSE: Breast self-examination
CBE: Clinical breast examination
PRISMA: Preferred Reporting Items for Systematic Reviews and Meta-Analyses
