# Supplementary material for: What determines the effects and costs of breast cancer screening? A protocol of a systematic review of reviews
Source: Syst Rev. 2017 Jun 28;6:122. doi: 10.1186/s13643-017-0510-y (PMC5490169; doi:10.1186/s13643-017-0510-y)
Supplement: Supplementary file 2 — The search strategy for Medline via PubMed. (DOCX 15 kb) [file 13643_2017_510_MOESM2_ESM.docx]

**Additional file 2. The search strategy for Medline via PubMed**

**The search strategy for PubMed in the review 1** (relevantly adapted to the other databases) includes the following combinations: 1 OR 2 OR 3 OR 4.

(1) (effectiveness OR adherence OR preferences OR compliance OR uptake OR guidelines OR efficacy OR attendance OR sensitivity OR specificity) AND breast cancer AND (screening OR mammography OR Ultrasonography OR Clinical breast examination OR Self-breast examination)).

(2) Breast Neoplasms[Mesh] AND Early Detection of Cancer[Majr];

(3) Breast Neoplasms [Mesh] AND Programme Evaluation/utilization[Mesh];

(4) Breast Neoplasms[Mesh] AND (Mammography[MeSH] OR Mass Screening[MeSH]).

**The search strategy for PubMed in the review 2** (relevantly adapted to the other databases) includes the following combinations: 1 OR 2 OR 3.

1. ((economic*[Title/Abstract]) OR cost*[Title/Abstract]) OR resource* [Title/Abstract]) OR hospitalization*[Title/Abstract]  OR absenteeism[Title/Abstract]  OR productivity [Title/Abstract]) AND breast cancer*[Title/Abstract].
2. Breast Neoplasms[Mesh] AND Costs and Cost Analysis[Mesh].
3. ((Health Resources [Mesh]) OR Health Resources/economics[Mesh] OR Health Resources/utilization[Mesh] ) AND Breast Neoplasms[Mesh].
