# Supplementary material for: What determines the effects and costs of breast cancer screening? A protocol of a systematic review of reviews
Source: Syst Rev. 2017 Jun 28;6:122. doi: 10.1186/s13643-017-0510-y (PMC5490169; doi:10.1186/s13643-017-0510-y)
Supplement: Supplementary file 3 — An adapted PRISMA checklist to assess transparency of presentation of the results in systematic reviews on costs and cost-effectiveness of breast cancer screening. (DOCX 15 kb) [file 13643_2017_510_MOESM3_ESM.docx]

**Additional file 3. An adapted PRISMA checklist to assess transparency of presentation the results in systematic reviews on costs and cost-effectiveness of breast cancer screening**

| **N** | **Location** | **Description** |
| --- | --- | --- |
| 1 | Title | Identify the report as a systematic review of cost, economic studies, cost-effectiveness studies or economic evaluations |
| **ABSTRACT** | | |
| 2. | Structured summary | Provide a structured summary including, as applicable: background; objectives; data sources; study eligibility criteria, participants, and interventions; study appraisal; results and results uncertainty; limitations; conclusions and implications of key findings. |
| **INTRODUCTION** | | |
| 3 | Rationale | Describe the rationale for the review in the context of what is already known as well as policy implications |
| 4. | Objectives | Provide an explicit statement of questions being addressed with reference to participants, interventions, comparisons, outcomes, and study design (PICOS). |
| **METHODS** | | |
| 5. | Protocol and registration | Indicate if a review protocol exists, if and where it can be accessed (e.g., Web address), and, if available, provide registration information including registration number. |
| 6. | Eligibility criteria | Specify study characteristics (e.g., PICOS, trial/model, duration of the study/model) and report characteristics (e.g., years considered, language, publication status) used as criteria for eligibility, giving rationale. |
| 7. | Information sources | Describe all information sources (e.g., databases with dates of coverage, contact with study authors to identify additional studies) in the search and date last searched. |
| 8. | Search | Present full electronic search strategy for at least one database, including any limits used, such that it could be repeated. |
| 9. | Study selection | State the process for selecting studies (i.e., screening, eligibility, included in systematic review, and, if applicable, included in the meta-analysis). |
| 10. | Data collection process | Describe method of data extraction from reports (e.g., piloted forms, independently, in duplicate) and any processes for obtaining and confirming data from investigators. |
| 11. | Data items | List and define all variables for which data were sought (e.g., PICOS, funding sources, study populations, model/trial based, perspective, sources of costs and outcomes) and any assumptions and simplifications made. |
| 12 | Risk of bias in individual studies | Describe methods used for assessing risk of bias (quality) of individual studies (including specification of whether this was done at the study or outcome level), and how this information is to be used in any data analysis and interpretation. |
| 13 | Main measures and sub-groups | State the principal summary measures and if any sub-groups analyses are conducted (if yes, provide a rationale). |
| **RESULTS** | | |
| 14 | Study selection | Give numbers of studies screened, assessed for eligibility, and included in the review, with reasons for exclusions at each stage, ideally with a flow diagram. |
| 15 | Study characteristics | For each study, present characteristics for which data were extracted (e.g., type of model or study size, PICOS, sensitivity, etc.) and provide the citations. |
| 16 | Risk of bias within studies | Present data on risk of bias/ quality assessment of each study and, if available, any outcome level assessment. |
| 17 | Results of individual studies | Present, for each study a simple summary data (values, ranges, references, and, if used, probability distributions, uncertainty, if applicable, incremental cost-effectiveness ratios). |
| 18 | Heterogeneity | If applicable, report variations among the studies in costs, outcomes, or cost-effectiveness. If relevant, provide explanations for variations (subgroups of patients with different baseline characteristics, variability in effects, etc.). |
| **DISCUSSION** | | |
| 19 | Summary of evidence | Summarize the main findings including the strength of evidence for each main outcome; consider their relevance to the decision makers |
| 20 | Limitations | Discuss limitations at study and at review-level (e.g., incomplete retrieval of identified research, reporting bias) and the generalizability of the findings and how the findings fit with current knowledge. |
| 21 | Conclusions | Provide a general interpretation of the results in the context of other evidence, and implications for future research. |
| **FUNDING** | | |
| 22 | Funding | Describe sources of funding for the systematic review and other support (e.g., supply of data); role of funders for the systematic review. |
